# Stability and Existence of Noncanonical I-motif DNA Structures in Computer Simulations Based on Atomistic and Coarse-Grained Force Fields

**DOI:** 10.3390/molecules27154915

**Published:** 2022-08-01

**Authors:** Tomasz Panczyk, Krzysztof Nieszporek, Pawel Wolski

**Affiliations:** 1 Jerzy Haber Institute of Catalysis and Surface Chemistry, Polish Academy of Sciences, ul. Niezapominajek 8, 30239 Cracow, Poland; pawel.wolski@ikifp.edu.pl; 2 Department of Theoretical Chemistry, Institute of Chemical Sciences, Faculty of Chemistry, Maria Curie-Sklodowska University in Lublin pl. Maria Curie-Sklodowska 3, 20031 Lublin, Poland; krzysztof.nieszporek@mail.umcs.pl

**Keywords:** i-motif, oxDNA, Martini, 3SPN, Amber, carbon nanotube

## Abstract

Cytosine-rich DNA sequences are able to fold into noncanonical structures, in which semi-protonated cytosine pairs develop extra hydrogen bonds, and these bonds are responsible for the overall stability of a structure called the i-motif. The i-motif can be formed in many regions of the genome, but the most representative is the telomeric region in which the CCCTAA sequences are repeated thousands of times. The ability to reverse folding/unfolding in response to pH change makes the above sequence and i-motif very promising components of nanomachines, extended DNA structures, and drug carriers. Molecular dynamics analysis of such structures is highly beneficial due to direct insights into the microscopic structure of the considered systems. We show that Amber force fields for DNA predict the stability of the i-motif over a long timescale; however, these force fields are not able to predict folding of the cytosine-rich sequences into the i-motif. The reason is the kinetic partitioning of the folding process, which makes the transitions between various intermediates too time-consuming in atomistic force field representation. Application of coarse-grained force fields usually highly accelerates complex structural transitions. We, however, found that three of the most popular coarse-grained force fields for DNA (oxDNA, 3SPN, and Martini) were not able to predict the stability of the i-motif structure. Obviously, they were not able to accelerate the folding of unfolded states into an i-motif. This observation must be strongly highlighted, and the need to develop suitable extensions of coarse-grained force fields for DNA is pointed out. However, it will take a great deal of effort to successfully solve these problems.

## 1. Introduction

Noncanonical DNA structures can be formed in many regions of the human genome [1,2,3], but the promoter regions, as well as telomeric and centromeric structures, are the most prone to the formation of DNA tetraplexes. The telomeric region of the human chromosome is built from highly repetitive sequences of bases. [4] The telomeric duplex consists of guanine-rich (G-rich; TTAGGG) and cytosine-rich (C-rich; CCCTAA) strands, and the single-stranded 3′ overhang is also built from G-rich sequences. The G-rich sequence can form a G-quadruplex in the presence of monovalent ions Na^+^ and K^+^ [5,6,7], while the C-rich strand can form an i-motif at slightly reduced pH when the formation of semi-protonated C:C^+^ pairs is possible [8,9].

These noncanonical structures have been extensively studied because their importance in biological processes is high and probably still not fully understood. The G-quadruplex is better recognized, and its role in the suppression of telomerase activity has been confirmed [6,7,8,9,10,11]. The biological role of the i-motif is still not fully understood, and there are some remarks that formation of the i-motif may lead to a similar effect as the G-quadruplex because it can indirectly induce G-quadruplex formation [10]. An interesting observation was selective induction of i-motif formation by carboxylated carbon nanotubes (CNTs) [12]. However, the i-motif is identified mainly as a cellular pH indicator or component of pH-triggered drug carriers [2,13,14].

Both noncanonical structures have been studied using molecular modeling [15,16,17] and atomistic force fields, usually belonging to the Amber family [18]. These atomistic force fields for DNA or RNA have continuously evolved since the 1990s [18]. Major refinements included a longer simulation timescale and high-level quantum chemical calculations, which resulted in the publication of ff99-bsc0 parametrization in 2007 [19]. That parametrization was found to be successful for the simulation of canonical DNA structures but led to some anomalies in the case of noncanonical structures, particularly those with loops [20]. Further major updates of Amber force fields for DNA were presented by two groups, leading to OL15 [21] and bsc1 [22] parametrizations. It is beyond the scope of this work to discuss these modifications in detail, but it is worth mentioning that OL15 focused on the refinements of ε/ζ and β dihedrals, while bsc1 increased the helical twist and yielded double-stranded DNA, hairpins, and G-quadruplexes in better agreement with experimental observations. One of the most impressive features of the Amber family of force fields, including the relatively old bsc0 parametrization, is their ability to predict unusual DNA structures for which parameterization was not carried out directly. These include hairpins, triplexes, quadruplexes, Z-DNA, and Hoogsteen duplexes [19].

The computational studies of G-quadruplexes and i-motifs have focused on the folded states, with the structural information taken from experiments; alternatively, the studies were involved in analysis of the unfolding processes of these structures. The modeling of folding processes starting, for example, from the coil or hairpin states has not been reported so far. This is because the formation of such high-symmetry structures in all-atom molecular dynamics simulations is very time-consuming, if at all possible. The problem resembles Levinthal’s protein-folding paradox which states that “*it would not be possible in a physically meaningful time to a protein to reach the native (functional) conformation by a random search of the enormously large number of possible structures*” [23]. That paradox has been formally solved by the statement that small energetic biases toward the target state reduce the conformational search to realistic folding times. However, the source of those small biases in standard unbiased computer simulations is unknown. In experiments, many factors may play a role, including, for example, the presence of chaperons [24], which, together with entropic factors coming from solvent, may construct the successful folding funnel.

Thus, in this work, we analyzed the stability and possible folding of a C-rich telomeric DNA fragment into an i-motif on the microsecond timescale using the Amber family of force fields for DNA. The intention of this analysis was not a direct observation of folding into the i-motif but rather a confirmation that such a transition cannot occur spontaneously despite the small size of this molecule and the very long computation time applied. We focused on the i-motif because this structure involves a proper description of protonated cytosines which represents, as we show, an additional difficulty in the application of coarse-grained force fields. We also paid attention to the effect of the carboxylated single-walled carbon nanotube as a factor which may facilitate the folding of a C-rich DNA fragment into an i-motif because similar observations have been reported in the literature [10,11,12,25].

Coarse-grained force fields are usually very helpful in accelerating large-scale transformations such as protein folding and micelle formation. The key point in coarse-graining is the reduction in the number of degrees of freedom and possible conformations in the folding pathway. Therefore, in this study we also focused on the application of these mesoscale computing methods using the most popular coarse-grained force fields for DNA (oxDNA [26,27], 3SPN [28,29], and Martini-DNA [30]) with the aim to obtain the folded i-motif-like structures.

All the studied approaches failed in the generation of a successful folding pathway of the C-rich DNA strand into an i-motif. This negative result of the performed research is important because it highlights the unresolved problems and encourages further research in this direction. The solution of this problem is not easy, as it requires either an application of adaptive sampling algorithms or a suitable extension of the coarse-grained force fields.

## 2. Results

### 2.1. Stability of I-Motif in the Amber Atomistic Force Field Representations

Both parametrizations of the Amber force field for DNA, i.e., bsc1 and OL15, were applied to study the behavior of a C-rich DNA fragment folded into a noncanonical i-motif structure. The analysis was focused mainly on the structural factors on a relatively long simulation timescale up to 800 ns. Some thermodynamic factors related to bsc1 parametrization were already addressed in our previous publications but using much shorter timescales [16,31,32]. The conclusions which were drawn from those studies were very clear; the i-motif spatial configuration is very stable with a deep free energy well only when half of the cytosines are protonated. Unprotonated cytosines lead to an unstable structure which unfolds spontaneously within a short time.

**Figure 1:**
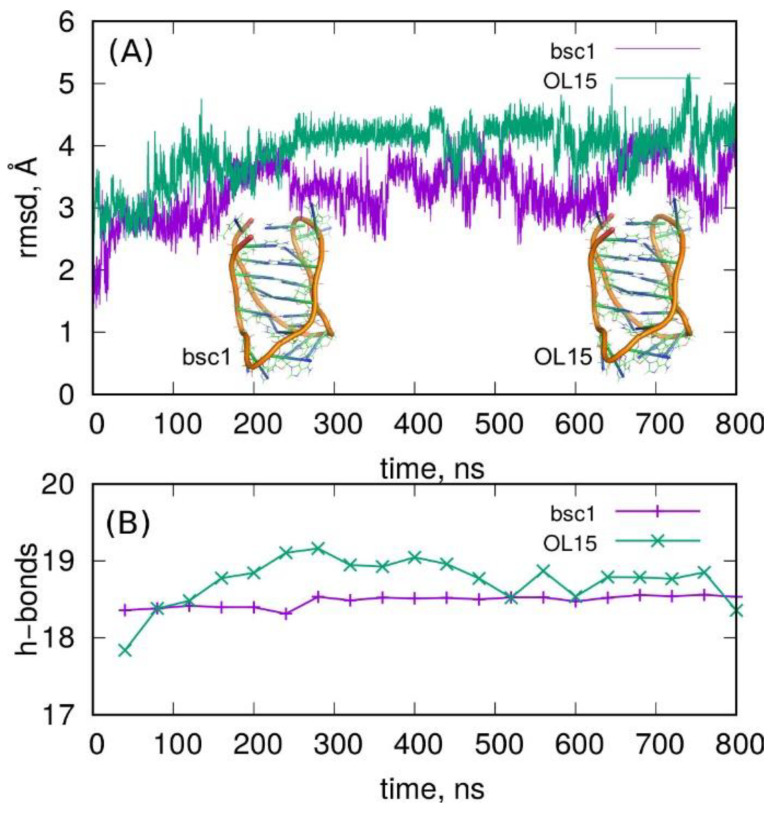
(**A**) Root-mean-square displacement (rmsd) of i-motif spatial structure for two parametrizations bsc1 and OL15 of the Amber force field. The snapshots show the structures of i-motifs in the last simulation frames for both parametrizations. (**B**) The number of hydrogen bonds between C:C^+^ residues obtained for both parametrizations.

**Figure 2:**
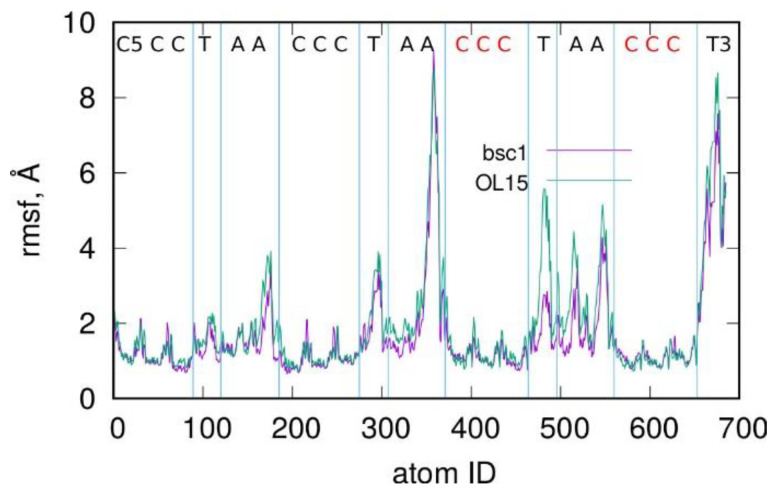
Root-mean-square fluctuation of atomic positions within the i-motif structure obtained for the studied parametrizations of the Amber force fields. The letters on the top show which residues the given atomic numbers belong to. The red C letters denote the protonated cytosines.

The essential results of the current long simulation timescale are shown in Figure 1 and Figure 2, where the root-mean-square displacement (rmsd), number of hydrogen bonds, and root-mean-square fluctuation (rmsf) are presented.

The plots of rmsd in Figure 1 with the initial i-motif structures as the reference states indicate that both parametrizations predicted stable i-motif conformations throughout the simulation. The rmsd values of ca. 3 Å are typical of thermal fluctuations. However, larger values about 4–5 Å, as observed for OL15 parametrization, indicated some deformation of the initial structure. Nevertheless, the rmsd in both cases did not reveal any growing tendency; hence, the i-motif was stable.

The rmsd plots in Figure 1 indicate that both parametrizations led to slightly different states of the i-motif structure. The OL15 generated structures slightly more different from the reference states (obtained from NMR analysis) than bsc1. Furthermore, analysis of the number of hydrogen bonds between C and C^+^ residues revealed significant differences between these two parametrizations. The bsc1 led to a very stable value close to 18 h-bonds which is the theoretically exact value. The OL15 revealed a significant drift of the number of h-bonds over time but remained close to 18. Therefore, the above observations indicate that OL15 parametrization led to a slightly more loose structure of the i-motif than bsc1.

**Figure 3:**
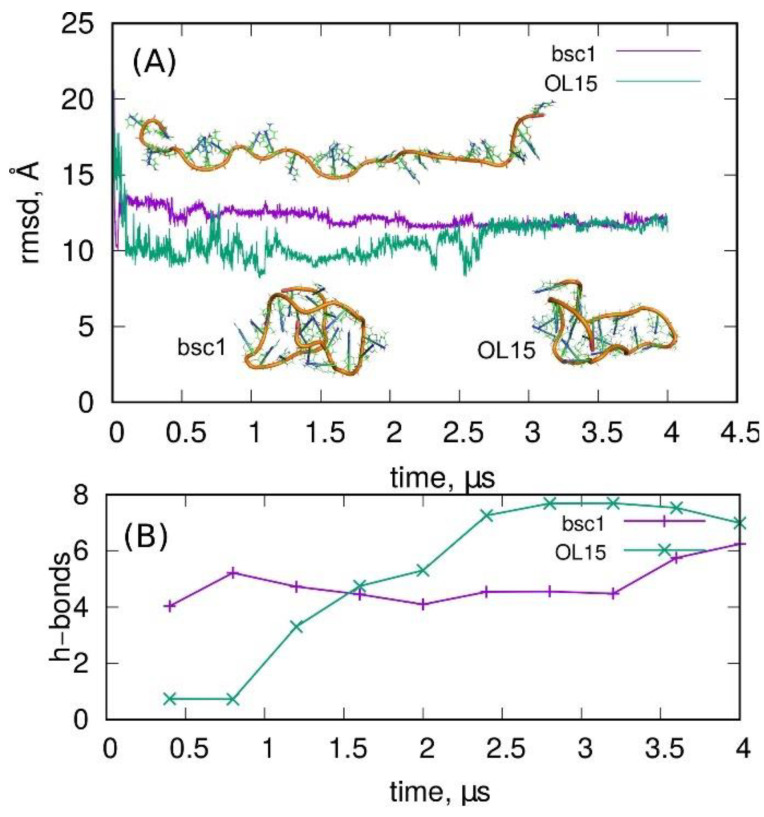
Root-mean-square displacement (rmsd) from the ideal i-motif structure for a C-rich sequence containing C:C^+^ pairs, i.e., ready to form the structured i-motif. The computations were carried out using two parameterizations of the Amber force field, i.e., bsc1 and OL15. The snapshot in the upper part of (**A**) shows the initial sequence in the random coil state, while the snapshots at the bottom are the final structures obtained after 4 μs simulation times. (**B**) The number of hydrogen bonds between C:C^+^ pairs during the simulation.

Figure 2 shows the fluctuations of atomic positions throughout the simulation, i.e., the root-mean-square fluctuation (rmsf). This plot informs about the mobility of individual atoms or residues (as indicated in the top part of the figure). We can conclude that the least mobile elements were C:C^+^ pairs, with the range of their fluctuations from the reference state remaining the same. This means that both parametrizations predicted strong binding in the semi-protonated cytosine pairs. The most mobile elements were adenine residues localized at the bottom part of the i-motif (see insets in Figure 1A) and 3′ thymine, but the range of their fluctuations remained the same for both parametrizations. The biggest differences were seen for thymine and adenines localized in the body of the i-motif. These bases were not stabilized by hydrogen bonds; thus, they revealed quite significant flexibility, and the differences between these two parametrizations became more visible.

The general observation is that both bsc1 and OL15 parametrizations predicted stable structures of the i-motif when built of semi-protonated C:C^+^ pairs. It seems that OL15 predicted a looser structure of the i-motif with larger displacement from the reference solution NMR structure. Additionally, the predictive features of the bsc1 and OL15 force fields must be strongly underlined; these force fields were not directly parametrized toward noncanonical DNA structures, but they perfectly describe them without additional assumptions or tweaking.

### 2.2. Folding of Cytosine-Rich Sequence into I-Motif

The stability of the i-motif spatial structure observed in Figure 1 suggests that the same C-rich DNA fragment, comprising C:C^+^ pairs, should spontaneously fold into an i-motif. Experimental data concerning the lifetimes of i-motifs at various pH and elevated temperatures suggest that melting of the i-motif takes several to several tens of seconds [4]. However, the kinetics of the reverse process has not been described, which suggests that the formation of i-motifs at temperatures lower than the melting temperature and at acidic pH is much faster. Molecular dynamics simulations are normally limited to nanosecond timescales; however, in the case of small systems and the utilization of GPU computing, the microsecond timescale is also achievable. Therefore, in this section, we present the results of our simulations aimed at observation of spontaneous (unbiased) folding of a C-rich telometric DNA fragment into an i-motif within the available microsecond timescale.

Looking at Figure 3, we can see that, after the initial rapid decrease in rmsd, it stabilizes at the value of 11–12 Å. There was no visible tendency of a further decrease in the rmsd, i.e., approaching the structure of an i-motif. The rmsd values above 10 Å indicate that the given structure was very far from the reference state; thus, we have to conclude that spontaneous formation of the i-motif did not occur over the considered very long timescale reaching 4 μs. Moreover, the absolutely flat shape of the rmsd observed for times greater than 2.5–3 μs suggests that the i-motif will never be formed spontaneously in the molecular dynamics simulations carried out within, let us say, a “reasonable” time. This is somewhat surprising because this particular system, though highly symmetric, is not very big. Nevertheless, the kinetic partitioning mechanism [33], with the appearance of multiple, well-separated, and structurally different conformational ensembles in the transition path, strongly slows down the formation of the i-motif structure. Experimental data suggest that it may take milliseconds [34], which remains too long to be effectively probed in molecular dynamics simulations. The structures shown in Figure 3 are probably representative of many possible intermediates located at the bottom of deep free energy wells, where spontaneous escaping from those wells is highly unlikely. Thus, continuation of the simulation to the mentioned millisecond timescale would, most probably, not lead to obtaining the i-motif structure. Interestingly, in an experimental flask, the analogous processes occur easily. Unfortunately, we cannot explain why such a paradox appears, but it is definitely similar to Levinthal’s protein folding paradox, as mentioned in Section 1.

The structures obtained in the 4 μs simulations using both parametrizations of the Amber force field are shown in Figure 3A. As can be seen, they were in the form of knots, which are structurally very different from the i-motif. Furthermore, analysis of hydrogen bonds between C:C+ residues shows that there were only 68 bonds, in contrast to the i-motif case with 18 h-bonds. Again, some interesting differences between bsc1 and OL15 parametrizations can be observed in Figure 3. Specifically, the bsc1 led to a quite gradual decrease in rmsd, which corresponds to folding of the initial structure into something like a hairpin. At the same time the number of h-bonds reached 4–6 and remained almost constant until the end of the simulation. The OL15 parametrization led to intense fluctuations of the structure. The rmsd quickly dropped to 10 Å but increased again to 11–12 Å. The number of h-bonds was almost zero until 1 μs and next grew to 8. Thus, the folding mechanisms of the C-rich DNA fragment differed significantly, but the final state was actually very similar for both parametrizations in terms of rmsd and the number of h-bonds.

A general conclusion coming from this part of the study is that atomistic force fields are unable to reproduce the formation of the i-motif within a microsecond timescale in an unbiased simulation. However, in some cases, such a folding process would be beneficial in modeling pH-dependent structural changes of various molecular structures containing cytosine-rich DNA sequences. Moreover, successful construction of such a set of restraints would formally correspond to the creation of a “folding funnel”, which was proposed as a physical explanation of Levinthal’s paradox. The problem is, however, explained by the physical sources responsible for the existence of such biasing forces in formally unbiased simulations. However, let us try to find a set of restraints which could speed up the formation of the i-motif in biased molecular dynamics simulations. The obvious choice is, of course, the application of moving restraints on the distances between N3 nitrogen atoms belonging to C:C^+^ pairs leading to the target distance of 2.8 Å. This set of six distances would generate hydrogen bonds responsible for the stability of the i-motif at acidic pH. However, it quickly turned out that this was not enough since the forces acting on these nitrogen atoms led to the formation of knots sterically blocking further folding to a structural i-motif.

**Figure 4:**
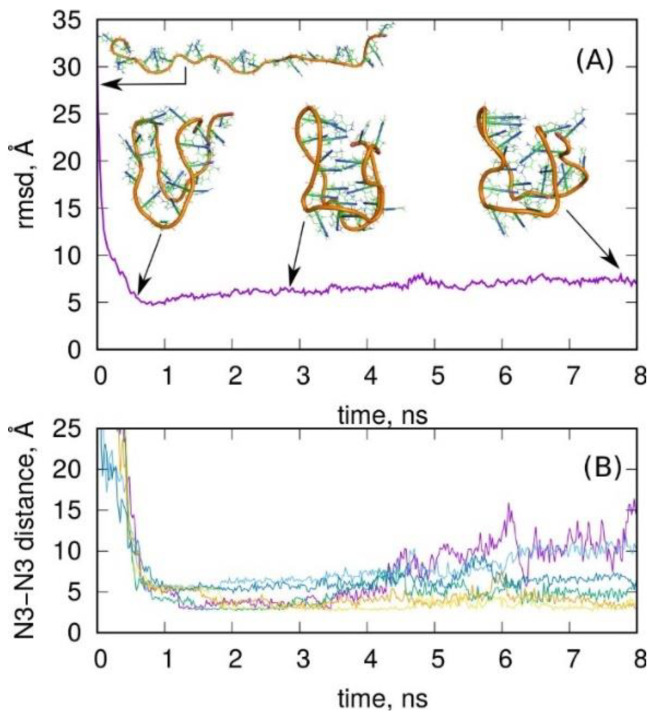
(**A**) The rmsd from the ideal i-motif structure obtained during enforced folding of the C-rich chain (the snapshot at the top of part (**A**)) into the i-motif form. The snapshots correspond to (from the left) loosely shaped i-motif-like structure obtained using forces acting on the backbone points, the structure obtained by applying the forces acting on N3 atoms in C:C^+^ pairs, and the structure obtained after releasing all the forces and allowing the structure to relax. (**B**) The distances between N3 atoms in C:C^+^ pairs during enforced folding of C-rich chain into i-motif like structure. The applied force field parametrization was the bsc1.

A better set of restraints was found after many trials, but its efficacy was still not satisfactory. The idea was to divide the folding process into two stages. The first stage was the formation of the loose shape of an i-motif-like structure by imposing five moving restraints onto distances between backbone points: (1) between terminal points of the whole chain: 13 Å, (2) and (3) between terminal points and the middle point of the chain: 20 Å, (4) between center of mass of terminal points and center of mass of points forming the bottom of i-motif: 28 Å, and (5) between middle points of the chain and center of mass of points forming the bottom of i-motif: 28 Å. The above set of restraints acted for some time (0.4 ns), enabling the formation of the loose shape (Figure 4A, snapshot on the left) of the i-motif from any initial structure. Afterward, these restraints were switched off, and the restraints enforcing the formation of hydrogen bonds between N3 atoms of C:C^+^ pairs were switched on, acting until 4 ns of simulation time. After that time, these forces were removed, and the system was allowed to relax. The whole process is illustrated in Figure 4.

Looking at the rmsd and six distances between N3 atoms, we can conclude that the forces imposed on the backbone points quickly led to the formation of a structure with relatively low rsmd from the i-motif, but with distances between N3 atoms very far from 2.8 Å. In the next step, when the new forces between N3 atoms were applied, we can see the significant reduction in those distances (some of them reached the values typical for hydrogen bonds), but the rmsd was not improved. The corresponding structure seems to be fairly similar to the i-motif shape. In the last step (t > 4 ns) the forces were switched off, and the structure was allowed to relax. However, the relaxation led to significant deterioration of the structure, as particularly seen in the distances between N3 atoms. Thus, the structure became actually similar to those described in Figure 3, suggesting that the formation of the i-motif is not possible on a reasonable timescale.

The conclusion is that the attempt to create a folding funnel failed. Of course, construction of a very detailed pathway involving many degrees of freedom which the system must follow due to the bias applied will finally lead to the assumed target structure. However, the system trajectory would then be artificial and the physical explanation of the existence of those biasing forces would be impossible whether in a simulation or in an experiment.

### 2.3. Folding of Cytosine-Rich Sequence in the Presence of a Carbon Nanotube

As already mentioned in Section 1, carboxylated single-walled carbon nanotubes were reported as i-motif-stabilizing agents [10,12,25]. In our previous studies, we addressed that problem but in the context of folding into an i-motif structure of unprotonated and protonated cytosine-rich sequences [16,32,35]. A general conclusion was that the protonated i-motif was stable when brought into contact with a carbon nanotube; however, the unprotonated structure, with an initial i-motif shape, deteriorated faster in the presence of a carbon nanotube compared to alone. Thus, the question is whether the unfolded sequence containing C:C^+^ pairs is able to fold spontaneously into an i-motif in the presence of a carboxylated single-walled nanotube. Such a mechanism is possible since the nanotube can act as a nucleation center for achieving the hairpin structure, followed by further folding into the tetraplex form.

**Figure 5:**
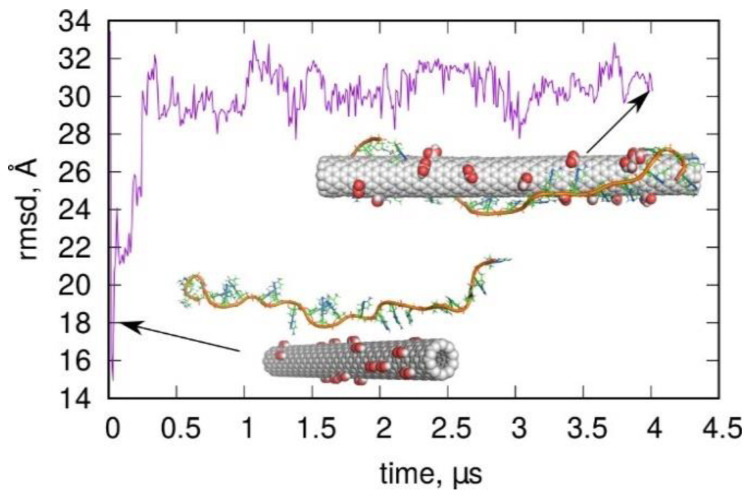
Evolution of cytosine-rich DNA fragment in contact with carboxylated carbon nanotube. The inset on the left shows the initial arrangement of the CNT and DNA, while the inset on the right shows the final state with DNA wrapped around the CNT. The calculations were performed using the bsc1 parametrization for DNA.

Figure 5 shows the results of such studies in the form of rmsd from the i-motif structure of the protonated cytosine-rich chain containing C:C^+^ pairs in a long 4 μs unbiased molecular dynamics simulation (using the bsc1 parametrization). The inset in Figure 5 shows the initial arrangement of the carboxylated nanotube and the DNA fragment. Therefore, they were initially separated and oriented almost perpendicularly. During the simulations, both species approached each other, and the DNA fragment started to wrap around the nanotube. The inset on the right of Figure 5 shows the final structure obtained after 4 μs. As can be seen, the wrapped form of the DNA fragment was the target structure, and it was very far from the i-motif shape, as indicated by the very large values of rmsd. The rmsd increased very quickly and reached a value of ca. 30 Å in less than 0.5 μs. This state did not change much in further stages of calculations and corresponded to the cytosine-rich chain wrapped around the nanotube. Thus, the carboxylated carbon nanotube did not help with folding into an i-motif; instead, it hindered such a process since the wrapped state of DNA seemed to be very stable.

### 2.4. Coarse-Grained Force Fields for DNA

#### 2.4.1. oxDNA Model

The oxDNA coarse-grained model, developed by Ouldridge et al. [26,27], provides a “top-down” approach in which the DNA components are treated as extended bodies, i.e., ellipsoids, and the interaction between them is represented as a set of only several interaction types. The model is, thus, very far from the atomistic description of the nucleotides but has been shown to correctly reproduce many different processes relevant to DNA nanotechnology, including origami nanostructures [36,37]. A further extension of the model led to the ability to reproduce minor and major grooves within the duplex, account for the variable ionic strength of a solution, and improve the description of the stability and closing rates of single-stranded DNA hairpins [27].

It is, therefore, reasonable to check if the oxDNA model is able to reproduce the stability of i-motifs or to speed up their formation on a coarse-grained scale. For that purpose, we prepared the input files for oxDNA simulations starting from the all-atom pdb file. The mapping of atomic coordinates to oxDNA beads can be performed using several tools, including easy-to-use web services such as TacoxDNA [38] and oxView [39]. Both services, however, failed to properly handle the names of protonated cytosines, and we had to either rename them (and, thus, lose information about their protonated state) or obtain input files with the protonated cytosines skipped. In the latter case, the oxDNA binary could not work with such incomplete input files.

**Figure 6:**
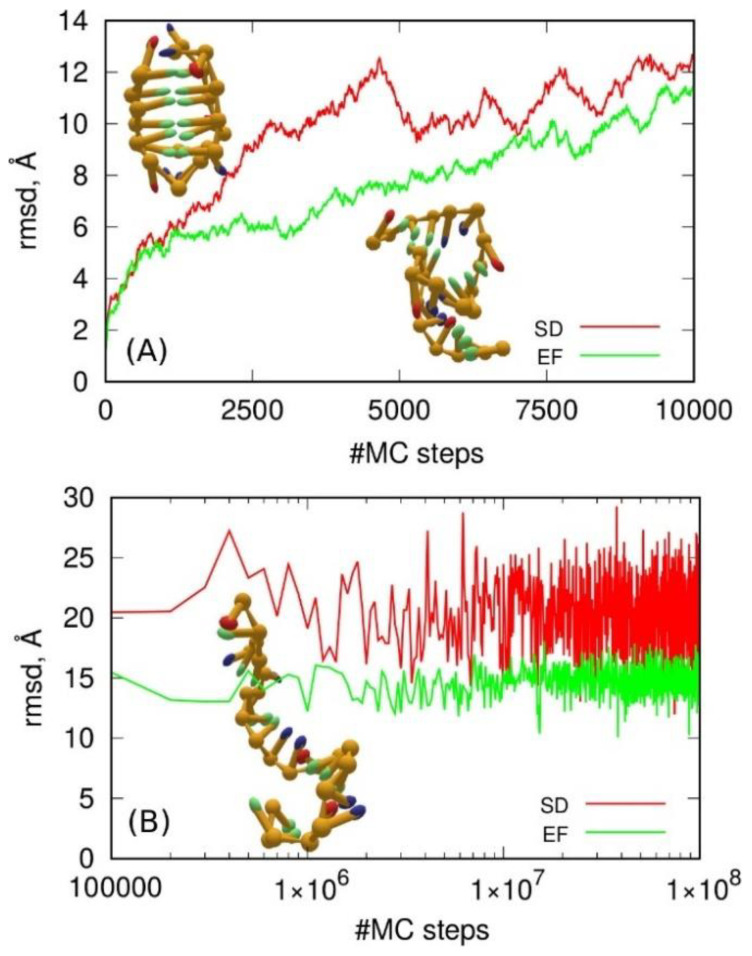
Root-mean-square displacement (rmsd) of the analyzed sequences from the initial i-motif structure obtained using Monte Carlo simulations according to the oxDNA2 model. (**A**) The initial stage up to 10,000 MC steps using the two approaches to base pairing, i.e., SD—sequence-dependent approach and EF—mutual traps between C:C^+^ pairs. Part (**B**) The whole simulation involving 10^8^ MC steps. The inset in (**B**) shows the final structure after 10^8^ steps for the SD approach, while the inset on the right of (**A**) shows the final structure obtained from EF approach. The rmsd values were calculated using the ideal i-motif structure (inset on the left of (**A**)) as a reference, and each configuration was aligned and best-fitted to the initial configuration. The trajectories of the oxDNA simulation were remapped into cartesian coordinates of the center of mass of the backbone and base beads using the tool traj2xyz.py provided in the oxDNA distribution package.

The oxDNA simulation can be carried out using molecular dynamics or the Monte Carlo method. Additionally, oxDNA2 offers the inclusion of a more realistic sequence-dependent stacking interaction, which is achieved by differentiating between the AA and TT stacking interaction strengths. It is also possible to impose an external force onto nucleotides using so-called “mutual traps”. The use of mutual traps can highly decrease the simulation time required by the folding of strands into target structures (e.g., DNA origami or DNA hairpins). Thus, we performed simulations of the i-motif according to these two approaches; the SD model used the sequence-dependent approach, while the EF model used the external forces between C:C^+^ pairs, which should form h-bonds within the i-motif. Of course, we had to treat the six protonated cytosines C^+^ within the i-motif as normal unprotonated ones due to the limitations of oxDNA parametrization. The obtained results are presented in Figure 6.

Observation of the simulation trajectories quickly led to the conclusion that the oxDNA model could not predict i-motif stability whether using the SD or the EF approach. As shown in Figure 6A the i-motif structure deteriorated continuously, and, within several thousands of MC steps, it became a random coil state. However, the difference between the SD and EF approaches is clear. The long-term behavior differed in terms of the rmsd values, which is not surprising since the EF model introduced artificial forces to keep the distances between C and C+ at ca. 10 Å. This, however, does not mean that the hydrogen bonds were preserved, but the whole structure was indeed more compact. Thus, the final conclusion is that the oxDNA coarse-grained model could not help in formation of noncanonical DNA structures in computer simulations.

#### 2.4.2. 3SPN Model

The three sites per nucleotide (3SPN) model proposed by Knotts et al. [28] represents a top-down approach for the description of nucleotides. In 3SPN, three sites are mapped onto the full atomistic representation of each base, sugar, and phosphate, and water is treated implicitly through Langevin dynamics [40]. In a further extension of the model, explicit ions were added [29], and the local molecular structure of the system under investigation was believed to be consistent with results from detailed atomistic representations.

In our exercises, we utilized the lammps [41] implementation of the 3SPN model distributed in the USER-3SPN package. The mapping of the all-atom configuration of the i-motif was achieved using the dedicated python tool pdb2cg_dna.py. Additionally, we generated a single-stranded DNA fragment with the same sequence as the i-motif but in a coiled state. Both models were subjected to MD simulations using an approach with explicit ions. It should be noted that the pdb2cg_dna.py tool was not able to handle the protonated cytosine residues. Thus, as before, in order to proceed, we switched to standard residue names, but lost information about the protonated state of these cytosine residues.

**Figure 7:**
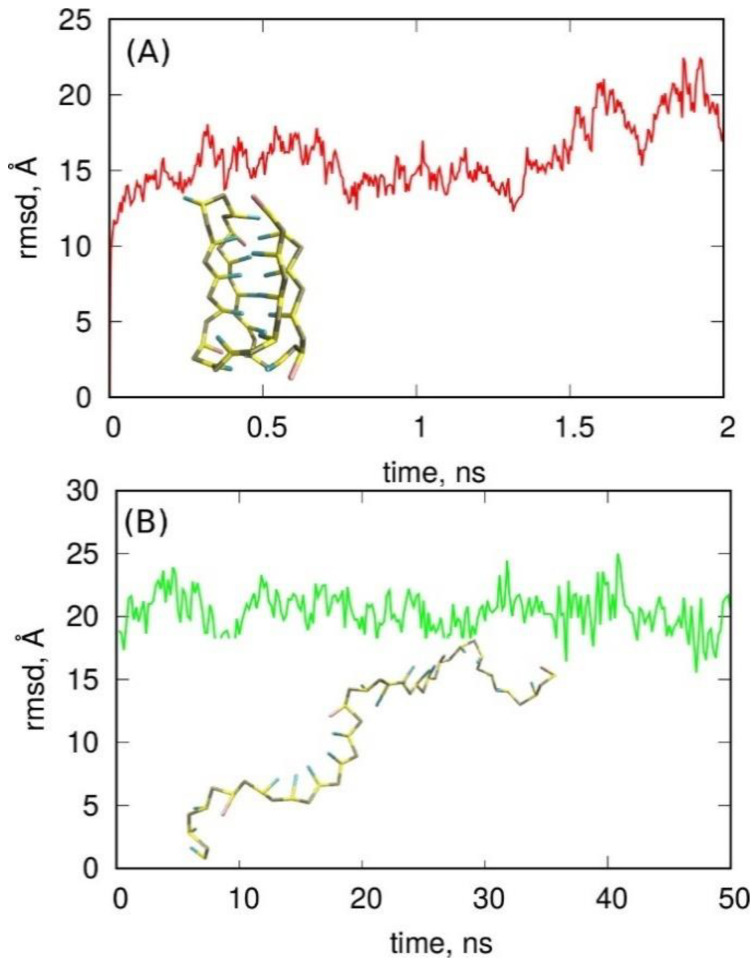
(**A**) Root-mean-square displacement (rmsd) of the analyzed structure from the ideal i-motif form in 3SPN representation (inset). (**B**) The rmsd from the ideal i-motif form but calculated for the single-stranded DNA fragment in the ideal coiled form. The snapshot in (**B**) shows the average final structure obtained from both (**A**,**B**) runs.

The obtained simulation results were analyzed mainly in terms of structural factors, using the rmsd with the i-motif structure as the reference. Figure 7 shows these results together with a graphical representation of the initial and the effective structures. As clearly seen in Figure 7A, the i-motif structure was unstable within the 3SPN representation. Within less than 0.5 ns, it completely deteriorated, as can be deduced from the rmsd values of approximately 15–20 Å. The inset in Figure 7A shows the initial i-motif structure in the 3SPN representation, while the inset in Figure 7B shows its state after about 2 ns of simulation. Figure 7B describes the results of the second exercise, which checked if the relevant fragment of single-stranded DNA was able to fold into an i-motif form over a longer timescale. As can be seen, the rmsd was very far from the reference value; thus, the conclusion is that this DNA fragment did not have the ability to form an i-motif within the 3SPN coarse-grained representation.

#### 2.4.3. Martini-DNA Model

Martini is probably the most popular general purpose coarse-grained force field [42]. Developed originally for lipid systems [43], it quickly became extended to all major groups of molecules, including DNA and RNA [30]. The mapping of an all-atom configuration into CG is quite standardized in Martini, as every four nonhydrogen atoms are merged into a single bead, while rings are merged into three atom fragments. In the particular case of DNA, the backbone is modeled using three beads by mapping the phosphate to one and the sugar to two beads. The bases are modeled as three-bead rings or four-bead rings. The model also assumes special bead parameters for modeling small and almost planar base pairs, while external forces are applied to include hydrogen bonding between complementary bases in double-stranded DNA. The model was tested and compared to results from atomistic force fields in the case of various single-stranded DNA fragments [30].

Preparation of input scripts for the i-motif was completed as described below. The fully atomistic structure of i-motif was used as the input for the martinize-dna.py python script with the ss (single-stranded) option for dnatype switch. The script, as usual, did not recognize protonated residues and skipped them in the output structure. Therefore, we had to rename the protonated cytosines as standard ones in order to proceed further, but lost the information about the protonated state of these bases. Next, the coarse-grained representation of the i-motif was used for the simulation box generation and solvation with water and ions.

Because the Martini parametrization is rather detailed and very likely to reproduce the stability of noncanonical DNA forms, we also checked the stability of the noncanonical DNA fragment complementary to the i-motif, i.e., the G-quadruplex. This structure does not require protonation of bases but only the presence of monovalent cations such as Na^+^. Indeed, we checked in our previous studies that the all-atom representation of the G-quadruplex was absolutely stable using the atomistic Amber bsc1 force field [44,45]. Thus, the all-atom pdb structure of G-quadruplex 2F8U, published by Dai et al. [46], was subjected to coarse-graining using the martinize-dna.py script with the same ss option for dnatype switch.

**Figure 8:**
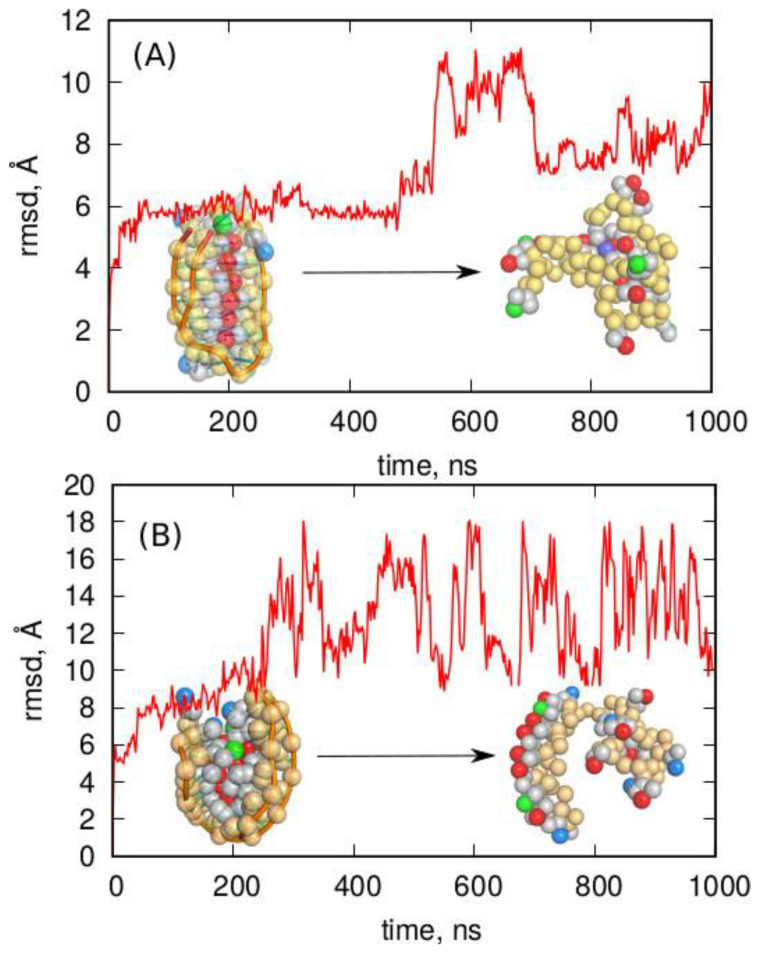
The rmsd determined for the i-motif (**A**) and G-quadruplex (**B**) in Martini coarse-grained representation. The snapshots on the left are the initial reference structures of both noncanonical DNA fragments, while the snapshots on the right are the corresponding structures at the end of the simulation.

Molecular dynamics simulations of both the i-motif and the G-quadruplex in Martini representation were carried out using gromacs [47] with standard settings for such calculations taken from the associated tutorials. The results of the simulations are presented in Figure 8 as the rmsd from the ideal coarse-grained form of the i-motif or G-quadruplex, plotted as a function of the simulation time.

Analysis of the results obtained using Martini led to clear conclusions. Specifically, neither of the noncanonical forms was stable in the Martini coarse-grained representation. In both cases, the initial structures deteriorated spontaneously within short times. Another conclusion is that the accepted loss of information about the protonation of the six cytosine residues in the i-motif was not the key reason for the failure of the Martini model. This is because deterioration also occurred for the G-quadruplex structure in which the mapping of the atomic structure into a coarse-grained one was straightforward without a loss of information on any factor.

**Figure 9:**
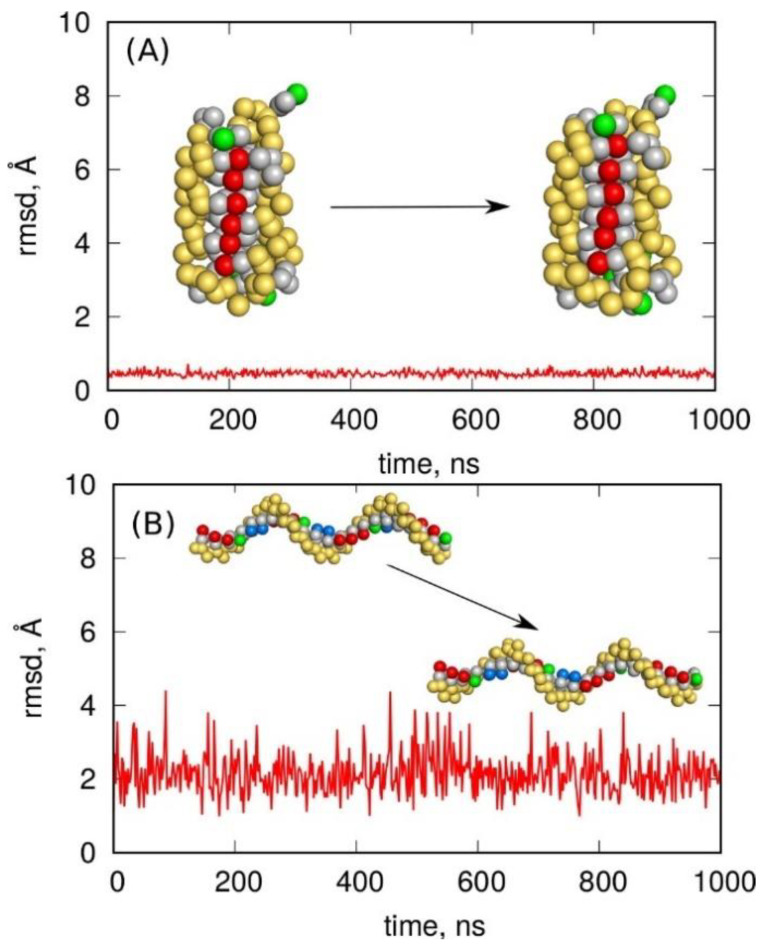
(**A**) The rmsd for the i-motif structure obtained using the stiff elastic network approach in a Martini coarse-grained model. (**B**) The rmsd for the same sequence of single-stranded DNA but starting from the ideal helix/coil form. The snapshots on the left show the starting/reference structures, while the snapshots on the right show the final structures at the end of the simulations.

The Martini representation also offers an extension of the force field using the concept of an “elastic network” [48]. This approach allows maintaining the canonical form of double-stranded DNA, but is also implemented for single-stranded DNA. Such an approach can formally reproduce any spatial form if the parameters of the elastic network are adequately tuned. However, we were mainly interested in the predictive features of the default settings which, for single-stranded DNA fragments, could be invoked using the “ss” or “ss-stiff” options in dnatype switch for single-stranded DNA when running the martinize-dna.py script. Therefore, the atomistic i-motif structure was subjected to coarse-graining while also using the concept of a stiff elastic network according to an analogous procedure to that described before. Furthermore, the G-quadruplex structure was subjected to coarse-graining with the option of a stiff elastic network applied. The obtained models for both noncanonical single-stranded DNA fragments were subjected to MD simulations, but the G-quadruplex model failed at the very beginning due to numerical instabilities. We, thus, conclude that the concept of stiff elastic networks applied to the spatial and densely packed G-quadruplex structure was physically incorrect. The same was probably true for the i-motif model; however, in this case, the calculations went smoothly. Nevertheless, the obtained results were not reliable, as shown in Figure 9.

As shown in Figure 9, the i-motif structure survived the whole 1000 ns simulation time period, which could suggest that the applied model correctly reproduced the noncanonical DNA form. However, looking at the values of rmsd (~0.5 Å) and their fluctuation, it is clear that the behavior of the model was unphysical. Simply, the rmsd never exceeded 0.5 Å, which is well below the 2–3 Å typical of thermal fluctuations in the atomistic model (See Figure 1). Moreover, the fluctuation of rmsd was very small, meaning that the structure was almost frozen. Thus, the stiff elastic network model generated an unphysical trajectory, and the apparent stability of the spatial structure of i-motif was a result of the strong external forces which overshadowed the true dynamics.

Figure 9B, in turn, addressed another problem, i.e., the ability of the C-rich telomeric single-stranded DNA sequence to fold into an i-motif within the applied stiff elastic network model. The starting structure was the (CCCTAA)n sequence in the form of a single helix/coil. The results of the calculations presented in Figure 9B led to the conclusion that the spatial structure of the chain did not change significantly (rmsd ~2Å), while the structure did not fold into an i-motif, hairpin, or even a random coil, which is a fast process even in fully atomistic simulations.

We, therefore, conclude that none of the studied coarse-grained models of DNA were able to correctly reproduce the i-motif structure. The results were either a maintenance of its shape or a reproduction of the spontaneous and fast folding of a coil/hairpin/random coil into the i-motif structure. The above conclusion seems to be trivial because none of these coarse-grained models were parametrized toward noncanonical DNA structures. However, the atomistic force fields (bsc1 or OL15) were able to correctly describe the structure of the i-motif despite not being parameterized toward such structures. Thus, the predictive features of the current parametrizations of the coarse-grained models are not good. However, the coarse-grained models seem to be tunable, and further optimization of their parameters can finally lead to next-generation models able to either reproduce the stability of noncanonical DNA forms or to observe the spontaneous folding to these forms.

## 3. Materials and Methods

The calculations were carried out according to two different approaches; the first was based on ab all-atom representation of the C-rich DNA fragment and focused on long-term simulations of spontaneous folding into the i-motif, whereas the second approach was based on the application of various coarse-grained models of DNA and an analysis of their ability to reproduce the spatial form of the i-motif with possible acceleration of the folding process. These two approaches utilized different methodologies and computational tools.

The all-atom simulations were based on the application of the Amber force field for DNA which is, as already discussed, available with two different parametrizations: bsc1 and OL15. The C-rich DNA was taken and adopted from the sequence d[CCCTA25mCCCTA2CCCUA2CCCT] published by Phan et al. [4,49] (pdb ID 1EL2). The pdb structure 1EL2 was modified by replacing 5mC with C and U with T, where C is cytosine, U uracil, and T is thymine. Because we were focused on the semi-protonated pairs C:C^+^, half of the cytosines were additionally replaced by their protonated counterparts. Hence, the C-rich i-motif sequence analyzed in this study was as follows: 5′–(CCCTAA)3CCCT–3′. It should be noted that a similar approach for the generation of an i-motif sequence was previously adopted by Smiatek et al. [15].

The all-atom molecular dynamics calculations were carried out using gromacs [47] software, and input files corresponding to the bsc1 and OL15 parametrizations were generated using the tleap program from the AmberTools16 package [50]. The conversion of Amber input files into gromacs files was achieved using the acpype script [51]. The simulation boxes contained ca. 15,000 molecules of water and suitable amounts of Na^+^ and Cl^−^ ions in order to neutralize the backbone charge and produce an ionic strength of the solution close to 0.15 M. The simulations were carried out at constant temperature (310 K) and constant pressure (1 atm), with periodic boundary conditions applied in all directions. The dimensions of the simulation box were ca. 7 × 7 × 10 nm. The electrostatic interactions were summed using the particle mesh Ewald approach.

In calculations involving the carbon nanotube, the methodology was slightly more complex. First, the structure of the carboxyl-functionalized CNT was generated using a self-designed script. The force field topology for functionalized CNT was generated using the antechamber program from the AmberTools16 and the acpype script, and the force field type was set to the general Amber force field (gaff) [52,53]. The partial charges on atoms comprising carboxyl groups were calculated using the RED Server Development service, which produces so-called resp charges [54]. We assumed that all 19 COOH groups (ca. 2% of the total number of C atoms) were non-protonated in the considered conditions. This is justified by the relatively large pKa of COOH groups linked to the CNT sidewall [55]. The nanotube chirality was (10,0); therefore, its diameter was ca. 7.5 Å, and the CNT length was ca. 100 Å. However, due to the periodicity applied to its structure, it was effectively infinitely long.

In calculations involving coarse-grained models of DNA, the methodology was adjusted to a given computational protocol. Accordingly, in the case of the oxDNA model, the calculations were carried out using standalone oxDNA software [26,27]. The preparation of input scripts and the analysis of output trajectories were performed using the oxView [39] service and TacoxDNA [38] web service. In the case of the 3SPN model of DNA, we utilized its lammps [41] implementation, whereas, for the Martini model, we applied gromacs software. Details and important settings related to running simulations with those coarse-grained models were provided in Section 2.4. The input files for all simulation variants are provided in Supplementary Materials.

## 4. Conclusions

The noncanonical i-motif DNA structure (and partly G-quadruplex) was studied in the context of its stability and possible formation using several computational approaches to DNA modeling. The atomistic representation of the i-motif within the Amber force field was successful. This means that the spatial structure of i-motif was intact over a relatively long timescale for both bsc1 and OL15 parametrizations. However, we found that fluctuations of either the atomic coordinates or the number of hydrogen bonds between C:C^+^ pairs differed significantly depending on the parameterization type. Generally, bsc1 led to a more static structure than OL15 in terms of rmsd and the number of hydrogen bonds. However, neither of the studied parametrizations was able to reproduce folding of the telomeric C-rich sequence into an i-motif. The 4 μs long unbiased simulations led to structures folded into knots or hairpins but equally far from the i-motif in terms of rmsd. Attempts to accelerate the formation of the i-motif using biased dynamics with moving restraints also failed, since the enforced i-motif-like structures were more prone to deterioration than to improving the spatial structure toward i-motif symmetry. The application of carboxylated carbon nanotubes as nucleation centers for folding of the C-rich sequence into an i-motif also failed, despite the literature data suggesting that carbon nanotubes can induce the formation of an i-motif in various conditions.

Further studies oriented mainly toward the application of coarse-grained force fields for DNA to study i-motif formation and stability led to further interesting conclusions. Specifically, the very popular oxDNA model was revealed to be unable to maintain the spatial structure of i-motif, and fast deterioration of the initial structure was observed. Obviously, the oxDNA model was unable to reproduce the folding of the C-rich sequence into the i-motif. Very similar behavior was revealed for the 3SPN coarse grained model, presenting deterioration of the structured i-motif and no ability to fold into an i-motif. The Martini model of DNA was applied to the i-motif and G-quadruplex, and it was found that both noncanonical forms were unstable in the Martini parametrization. The application of a stiff elastic network approach in the Martini representation of the i-motif led to rather nonphysical behavior with very low rmsd fluctuations and a frozen state of the i-motif spatial structure.

Generally, we can conclude that there are no good tools for modeling i-motif formation in molecular dynamics simulations. Atomistic force fields are not robust enough to effectively probe the whole configurational space and spontaneously find the configuration of the i-motif. The coarse-grained force fields are not able to adequately map the protonated nucleic acids into their beads. Furthermore, the coarse-grained force fields in their standard representations are not able to properly describe Hoogsteen hydrogen bonds or stacking interactions in the case of noncanonical forms. However, the coarse-grained force fields usually provide an option for extension of their capabilities. Thus, it is strongly needed to perform an update of the current coarse-grained parametrizations in order to equip them with the ability to describe noncanonical DNA forms and to observe folding of the corresponding DNA sequences into noncanonical structures.

## Data Availability

The atomistic structures of the i-motif, G-quadruplex, and unfolded i-motif used in the all-atom simulations and as inputs for mapping into coarse-grained models are available as pdb (Protein Data Bank) files in the Supplementary Materials as imotif.pdb, gquadruplex.pdb, and ccctaa.pdb, respectively. The gromacs version was 2020.6, https://ftp.gromacs.org/gromacs/gromacs-2020.6.tar.gz (accessed on 20 September 2021), and the input files for the simulation of folding with bsc1 and OL15 parametrizations are available as bsc.gro, bsc.top, ol.gro, and ol.top files, which were generated using tleap from AmberTools16, https://ambermd.org/, (accessed on 7 December 2016) and AcPyPE, https://github.com/alanwilter/acpype, (accessed on 19 January 2017). The carboxylated carbon nanotube atomic structure was generated using a self-designed script and is available as a gromacs input file:cnt.gro, while its force field topology file was generated as a general Amber force field (gaff) file using AcPyPE and is available as a cnt.itp gromacs input file. The 3SPN coarse-grained files for the i-motif are available as conf_lammps.in and in.run input files for lammps. The lammps version was 7 August 2017, https://download.lammps.org/tars/lammps-7Aug2019.tar.gz, (accessed on 25 February 2022) because this is the latest version which can be built using the USER-3SPN package for coarse-grained DNA simulations. The oxDNA input files were generated using the oxView https://sulcgroup.github.io/oxdna-viewer/ (accessed on 3 March 2022) and TacoxDNA http://tacoxdna.sissa.it/ (accessed on 3 March 2022) web services and are available as oxdna.input.top and oxdna.input.dat files. These files were used in oxDNA standalone code, obtained from https://sourceforge.net/projects/oxdna/files/oxDNA_2.4_RJUNE2019.tgz (accessed on 3 March 2022) and run using two settings files, oxdna.imotif_seq_dep and oxdna.imotif_trap, for the sequence-dependent and mutual traps simulations, respectively. The Martini-DNA input files were generated using the martinize-dna.py script distributed on the Martini web page http://cgmartini.nl/index.php/force-field-parameters/dna (accessed on 14 March 2022). The obtained input files for gromacs are available as martini.imotif.top, martini.imotif.gro, martini.gq.top, and martini.gq.gro for the i-motif and G-quadruplex, respectively. All files are available in the Supplementary Materials.

## Supplementary Materials

The following supporting information can be downloaded at https://www.mdpi.com/article/10.3390/molecules27154915/s1: The input files for all simulation variants, mentioned under Data and Software Availability, are provided.

## Sample Availability

Samples are available from the authors.
